# Supplementary material for: Brain functional networks associated with social bonding in monogamous voles
Source: eLife. 2021 Jan 14;10:e55081. doi: 10.7554/eLife.55081 (PMC7847304; doi:10.7554/eLife.55081)
Supplement: Transparent reporting form [file elife-55081-transrepform.docx]

***eLife’s* transparent reporting form**

We encourage authors to provide detailed information *within their submission* to facilitate the interpretation and replication of experiments. Authors can upload supporting documentation to indicate the use of appropriate reporting guidelines for health-related research (see [EQUATOR Network](http://www.equator-network.org/%20)), life science research (see the [BioSharing Information Resource](https://biosharing.org/)), or the [ARRIVE guidelines](http://www.plosbiology.org/article/info:doi/10.1371/journal.pbio.1000412) for reporting work involving animal research. Where applicable, authors should refer to any relevant reporting standards documents in this form.

If you have any questions, please consult our Journal Policies and/or contact us: [editorial@elifesciences.org](mailto:editorial@elifesciences.org).

**Sample-size estimation**

- You should state whether an appropriate sample size was computed when the study was being designed
- You should state the statistical method of sample size computation and any required assumptions
- If no explicit power analysis was used, you should describe how you decided what sample (replicate) size (number) to use

Please outline where this information can be found within the submission (e.g., sections or figure legends), or explain why this information doesn’t apply to your submission:

Thirty-two prairie voles (*Microtus ochrogaster*), 16 males and 16 females, were used in the study. The number of subjects evaluated for each experiment and its subsequent analysis are reported in the text and figure legends. The number of subjects per group is comparable to the largest sample sizes using rsfMRI in rodents (Bajic et al., 2016; Christiaen et al., 2019; Grandjean et al., 2014). Sample-size estimation is described in the “Animals” section of Materials and Methods.

**Replicates**

- You should report how often each experiment was performed
- You should include a definition of biological versus technical replication
- The data obtained should be provided and sufficient information should be provided to indicate the number of independent biological and/or technical replicates
- If you encountered any outliers, you should describe how these were handled
- Criteria for exclusion/inclusion of data should be clearly stated
- High-throughput sequence data should be uploaded before submission, with a private link for reviewers provided (these are available from both GEO and ArrayExpress)

Please outline where this information can be found within the submission (e.g., sections or figure legends), or explain why this information doesn’t apply to your submission:

Due to the longitudinal nature of the experiment, each subject underwent three MRI acquisition sessions in different time points. More details are described on the “Image acquisition” section of Materials and Methods. In behavioral experiments, animals were tested only once for initial cohabitation, once for partner preference test, and once as stimuli for their partner in the partner preference test to avoid unwanted habituation-familiarization processes. No outliers were identified, however, two rsfMRI datasets of two different subjects were discarded for not having the necessary quality. The management of MRI missing data in some subjects is specified in the “Functional connectivity analysis” section of Materials and Methods.

**Statistical reporting**

- Statistical analysis methods should be described and justified
- Raw data should be presented in figures whenever informative to do so (typically when N per group is less than 10)
- For each experiment, you should identify the statistical tests used, exact values of N, definitions of center, methods of multiple test correction, and dispersion and precision measures (e.g., mean, median, SD, SEM, confidence intervals; and, for the major substantive results, a measure of effect size (e.g., Pearson's r, Cohen's d)
- Report exact p-values wherever possible alongside the summary statistics and 95% confidence intervals. These should be reported for all key questions and not only when the p-value is less than 0.05.

Please outline where this information can be found within the submission (e.g., sections or figure legends), or explain why this information doesn’t apply to your submission:

Statistical methods used in the study are described in detail in the “Statistical analysis” section of Materials and Methods. All p values are reported exact in the text and APA statistical reporting guidelines were followed. Whenever possible, effect size and confidence intervals were reported. For better visualization, statistically significant p values may be reported in figures as *= p<0.05, **= p<0.01, ***= p<0.001. Data in the study is presented as mean ± standard error of the mean unless otherwise noted.

(For large datasets, or papers with a very large number of statistical tests, you may upload a single table file with tests, Ns, etc., with reference to sections in the manuscript.)

**Group allocation**

- Indicate how samples were allocated into experimental groups (in the case of clinical studies, please specify allocation to treatment method); if randomization was used, please also state if restricted randomization was applied
- Indicate if masking was used during group allocation, data collection and/or data analysis

Please outline where this information can be found within the submission (e.g., sections or figure legends), or explain why this information doesn’t apply to your submission:

Group allocation is described in the “Animals” section of Materials and Methods. For the cohabitation protocol and behavioral experiments, animals were assigned randomly with specified restrictions applied. Details are described in the “Cohabitation and behavior analysis” and “Partner Preference Test” sections of Materials and Methods.

**Additional data files (“source data”)**

- We encourage you to upload relevant additional data files, such as numerical data that are represented as a graph in a figure, or as a summary table
- Where provided, these should be in the most useful format, and they can be uploaded as “Source data” files linked to a main figure or table
- Include model definition files including the full list of parameters used
- Include code used for data analysis (e.g., R, MatLab)
- Avoid stating that data files are “available upon request”

Please indicate the figures or tables for which source data files have been provided:

Source numerical data for Figure 2, Figure 3, Figure 4, Figure 5 and Figure 6 are included. Code for Figure 5 is available at https://cran.r-project.org/web/packages/NBR/index.html. Resting-state functional magnetic resonance imaging (rsfMRI) preprocessed data series of all subjects used in the study are uploaded in Alcauter, Sarael et al. (2020), Data from: Brain functional networks associated with social bonding in monogamous voles, v2, Dryad, Dataset, https://doi.org/10.5061/dryad.1rn8pk0q9
